# Proceedings of the State Medical Society

**Published:** 1855-01

**Authors:** 


The Proceedings of the State Medical Society.—We have been looking anxiously for these proceedings, and hope that the Secretary will be able to present them to the public before long. If the delay in the publication arises from a deficiency in the Treasury, we trust and hope for the honor of the members of the society, it will soon be supplied. Let the Secretary call upon the delinquents, who, we trust, will have no hesitation in forwarding their annual tribute.

